# Effects of Insemination Quantity on Honey Bee Queen Physiology

**DOI:** 10.1371/journal.pone.0000980

**Published:** 2007-10-03

**Authors:** Freddie-Jeanne Richard, David R. Tarpy, Christina M. Grozinger

**Affiliations:** 1 Department of Entomology, North Carolina State University, Raleigh, North Carolina, United States of America; 2 Department of Genetics, North Carolina State University, Raleigh, North Carolina, United States of America; 3 W.M. Keck Center for Behavioural Biology, North Carolina State University, Raleigh, North Carolina, United States of America; The University of New South Wales, Australia

## Abstract

Mating has profound effects on the physiology and behavior of female insects, and in honey bee (*Apis mellifera*) queens, these changes are permanent. Queens mate with multiple males during a brief period in their early adult lives, and shortly thereafter they initiate egg-laying. Furthermore, the pheromone profiles of mated queens differ from those of virgins, and these pheromones regulate many different aspects of worker behavior and colony organization. While it is clear that mating causes dramatic changes in queens, it is unclear if mating number has more subtle effects on queen physiology or queen-worker interactions; indeed, the effect of multiple matings on female insect physiology has not been broadly addressed. Because it is not possible to control the natural mating behavior of queens, we used instrumental insemination and compared queens inseminated with semen from either a single drone (single-drone inseminated, or SDI) or 10 drones (multi-drone inseminated, or MDI). We used observation hives to monitor attraction of workers to SDI or MDI queens in colonies, and cage studies to monitor the attraction of workers to virgin, SDI, and MDI queen mandibular gland extracts (the main source of queen pheromone). The chemical profiles of the mandibular glands of virgin, SDI, and MDI queens were characterized using GC-MS. Finally, we measured brain expression levels in SDI and MDI queens of a gene associated with phototaxis in worker honey bees (*Amfor)*. Here, we demonstrate for the first time that insemination quantity significantly affects mandibular gland chemical profiles, queen-worker interactions, and brain gene expression. Further research will be necessary to elucidate the mechanistic bases for these effects: insemination volume, sperm and seminal protein quantity, and genetic diversity of the sperm may all be important factors contributing to this profound change in honey bee queen physiology, queen behavior, and social interactions in the colony.

## Introduction

While many previous studies have considered the effects of mating on the physiology and behavior of female insects (i.e., reference 1), the effects of mating number and semen quantity have not been broadly considered. Honey bees are an excellent system in which to study this process: colonies typically consist of a single reproductive queen and thousands of sterile worker bees, and the queen mates with several males (12 males on average; reviewed in reference 2) during a brief period in her early adult life. However, studies of the effects of mating number have focused almost exclusively on the consequences of worker genetic diversity on overall colony health [3]–[5] and social interactions [6]. Relatively little is known about how queen mating number may alter queen physiology and thereby alter intracolony social interactions. Understanding the physiological effects of multiple mating could offer insights into the mechanisms that govern mating behavior and how the process is regulated in honey bees and female insects in general, as well as what consequences this may have for colony organization in bees and other social insects.

Virgin honey bee queens initiate mating very early in their lives, when they are approximately 1–2 weeks old, by taking multiple mating flights and mating with numerous males (drones) on each flight. On average, queens are successfully inseminated by 12 males (based on molecular genotyping of workers [2]), but mating number is highly variable among queens (range from 1 to 28). The factors that determine mating frequency are not fully understood [7], [8]. Mating has profound and permanent effects on queen behavior, physiology, and resultant queen-worker interactions. Once they begin to oviposit, mated queens will never mate again and will remain in the colony for the rest of their lives (unless they depart during colony swarming). Mating stimulates vitellogenesis and oocyte-maturation in the ovaries [9], which prompts the initiation of egg-laying of up to 1500 eggs/day [10]. There are also profound changes in a queen's brain after mating, where levels of dopamine significantly decrease [11] and the ratio of the neuropil/cell body volume in the mushroom bodies significantly increases [12].

Finally, mated and virgin queens differ dramatically in their pheromone profiles, and these pheromones are important for regulating colony organization and worker behaviour [13], [14]. Queens produce pheromones from a variety of glands, and the complete “queen pheromone” has not been fully characterized [15]–[17]. However, five components in particular, termed “queen mandibular pheromone” or QMP, are produced by the mandibular gland and elicit many similar worker behavioral and physiological responses as a live queen. QMP consists of 9-oxo-(*E*)-2-decenoic acid (ODA), (*R*)- and (*S*)-9-hydroxy-(*E*)-2-decenoic acid (9-HDA), methyl *p*-hydroxybenzoate (HOB), and 4-hydroxy-3-methyoxyphenylethanol (HVA) [18]. QMP has both primer (long-term) and releaser (short-term) effects on worker behaviour and physiology. It elicits a “retinue response”, in which workers are attracted from a distance (several cm) and then antennate and groom the queen [18]–[21]. QMP also inhibits worker ovary activation [22], [23], inhibits queen cell production by workers [24], [25], stimulates pollen foraging and brood rearing in small, newly founded colonies [26], increases nectar foraging [27], [28], and delays the age-at-onset of foraging and reduces juvenile hormone secretion [27]. Some components of QMP are involved in drone attraction and mating [29], [30]. However, while the five component QMP blend is a potent modulator of worker behaviour and physiology, it is still not as potent as a live queen, suggesting that additional pheromonal components may exist. Indeed, four additional compounds were identified that synergize with QMP to increase the retinue response, but even those nine compounds do not elicit as robust a response as a live queen [17].

Although it is clear that mandibular gland pheromone profiles differ dramatically between young virgins and naturally mated, laying queens, the short-term effects of mating on pheromone profiles are largely unknown. Plettner et al. [14] compared the quantities of the QMP components between 6-day-old virgins and 1-year-old mated laying queens, and found that mated queens had significantly higher levels of 9-ODA, 9-HDA, HOB and HVA than did virgins. However, a similar study by Slessor et al [13], which compared 6- and 12-day-old virgins with mated queens that had been laying for 1 day, 5 weeks, or 2 years, found different results. In that study, levels of 9-ODA were not significantly different between any of these groups, 9-HDA levels were significantly higher in mated versus virgin queens, HOB levels were significantly higher in the 2-year-old mated queens than in both the virgins and 1-day laying queens and intermediate in the 5-week laying queens, and HVA levels were significantly higher in the 2-year mated queens compared to all other groups. Thus, pheromone profiles are strongly affected by mating, age (or perhaps time since mating), and potentially by egg-laying. Finally, Apsegaite and Skirkevicius [31] found slight differences in the quantity of 9-ODA between different strains of honey bees, suggesting that genotypic differences might also alter pheromone profiles.

Given the highly variable mating number and the profound effects mating has on queens, we hypothesize that mating number could modulate queen physiology, behavior, and pheromone production. However, “mating number” comprises a host of factors, including the number and duration of mating flights, the physical act of insemination, the volume of ejaculate, the quantity and viability of sperm and seminal proteins in the ejaculate, and the genetic diversity of sperm and seminal proteins. Furthermore, given that queens mate during flights several meters above the ground, it is not possible to control natural mating behavior. However, the number of males that inseminate a queen can be precisely controlled by using instrumental insemination [32]. Previous studies have demonstrated that queens inseminated with lower quantities of semen (less than 8 µL) are more likely to continue to take mating flights [33], but the effects of insemination quantity on other aspects of queen physiology have not been considered. To begin to address these questions, we focused on single-drone inseminated (SDI, total semen from one drone) and multi-drone inseminated (MDI, total semen from 10 drones) queens. We monitored attraction of the workers to SDI or MDI queens in observation hive colonies, as well as the attraction of workers to virgin, SDI, and MDI queen mandibular gland extracts in cages. The chemical profiles of the mandibular glands of virgin, SDI, and MDI queens were characterized using GC-MS. Finally, since fully inseminated queens cease to be phototactic and no longer take mating flights, we measured brain expression levels in SDI and MDI queens of a gene that is associated with phototaxis in worker honey bees (*Amfor*, the foraging gene; [34].

## Results

### Behavioral assays

#### Observation hives

**Figure 1:**
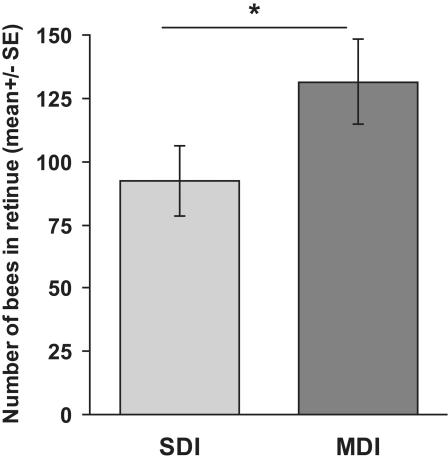
Workers are more attracted to MDI than SDI queens in observation hives. Following insemination, the retinue response to SDI and MDI queens was monitored in observation hives, twice a day for 5 days. MDI queens attracted significantly more worker bees in their retinue than the SDI queens did (F = 6.73, p = 0.02). (SDI: 7 queens; MDI: 5 queens).

One of the most measurable effects of queen pheromone is the induced retinue response, in which workers are attracted to the queen from a short distance, and lick and antennate her. Following insemination, the retinue response to SDI and MDI queens was monitored in observation hives, twice a day for 5 days. MDI queens attracted significantly more worker bees in their retinue than the SDI queens (F = 6.73, p = 0.02; Figure 1). There was no significant effect of day (F = 0.46, p = 0.76) or day*treatment interaction (F = 0.68, p = 0.61).

#### Cage assays

**Figure 2:**
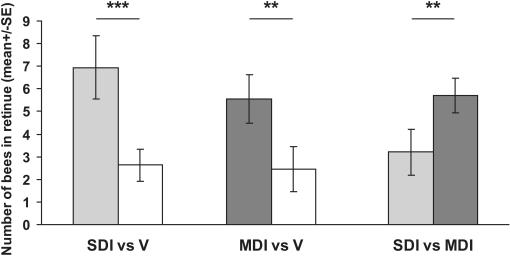
Workers are more attracted to the mandibular gland extracts of MDI than SDI queens. Caged workers were exposed to mandibular gland extract of two different queens simultaneously, and the retinue response to each extract was monitored. Workers were significantly more attracted to the extracts of inseminated versus virgin queens, and significantly more attracted to the extracts of MDI versus SDI queens (0.05 queen equivalents: Virgin vs SDI queens (n = 19); Virgin vs MDI (n = 20). MDI vs SDI (n = 21); all comparison with t-test: t<2.7, p<0.01). ** p<0.01 and *** p<0.001

Worker retinue responses to the mandibular gland extracts of virgin, SDI, and MDI queens were tested in cages containing 4-day-old bees. The retinue size was equivalent whether workers were exposed to virgin, SDI, or MDI queen mandibular gland extracts (H(2,18) = 0.615 p = 0.735; data not shown). However, worker bees exposed to two different mandibular gland extracts at the same time preferred gland extracts from SDI and MDI queens to virgins (respectively: t = 4.2, df = 36, p<0.001 and t = 3.1, df = 38, p<0.01, Figure 2), and preferred MDI extracts to SDI extracts (t = 2.7, df = 40, p<0.01). Data for the preference assay represent two trials which were not significantly different and were pooled for the subsequent analysis.

##### Chemical analysis of queen mandibular gland profile

**Figure 3:**
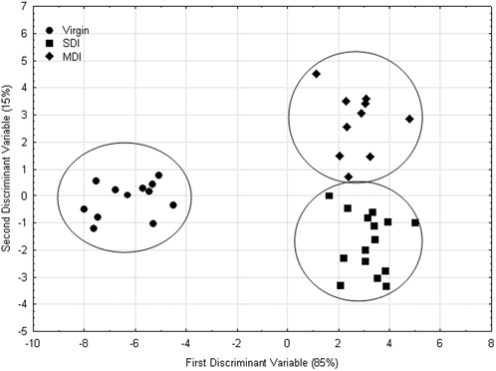
Insemination quantity significantly alters the chemical profile of mandibular glands. Chemical composition of the mandibular gland extracts of Virgin, SDI and MDI queens were analysed using gas-chromatography. Discriminant analysis of mandibular extract of virgin, SDI, and MDI queens was based on the relative proportion of the chemical compounds (F(33, 36) = 9.33. p<10^−4^; All group distances <0.005). Ellipses have been drawn to emphasize the categories, but have no specific statistical meaning

The chemical composition of the mandibular gland extract of virgin, SDI, and MDI queens were analysed using gas chromatography. A total of 27 compounds were apparent in the GC analysis and used in the subsequent comparisons. There were significant differences in the overall chemical profiles between the three groups of queens, as revealed by a discriminant analysis (F(33, 36) = 9.33, p<10^−4^; Figure 3). Two discriminant variables explained 100% of the variation. The chemical profiles of the virgin queen mandibular glands were most different from the two groups of mated queens (85% of the variation), and the other 15% of the variation could be attributed to insemination quantity.

Next, when the actual quantities of the compounds are analyzed (Table 1), mated queens had significantly lower levels of 18 compounds relative to virgins, and higher levels of one compound. MDI queens had significantly lower levels of seven compounds than SDI queens, and significantly higher levels of two compounds. Of the five QMP components, the quantities of 9-ODA, 9-HDA, and HVA were all significantly lower in mated queens compared to virgins. Levels of 9-ODA and 9-HDA were also significantly lower in the MDI queens than in the SDI queens.

**Table 1:** Absolute quantity of the mandibular gland compounds (mean±STD) in µg of virgin, SDI and MDI queens.

	Retention Index	Virgin (N = 12)		Inseminated (n = 25)		Mann Whitney (Virgin vs Inseminated)		SDI (n = 15)		MDI (n = 10)		Mann Whitney (SDI vs MDI)	
		mean	STD	mean	STD	*U*	p	mean	STD	mean	STD	*U*	p
Unidentified 1	1408	0.70	±0.09	0.70	±0.04	143	0.835	0.69	±0.04	0.71	±0.09	69	0.765
HOB	1499	0.20	±0.01	0.20	±0.02	126	0.451	0.22	±0.03	0.16	±0.04	49	0.160
4-hydroxy-3methoxybenzoic acid	1512	4.58	±0.44	2.32	±0.20	31	**0.000**	2.70	±0.21	1.74	±0.33	34	**0.023**
8-HOAA	1627	12.12	±1.08	6.19	±0.80	42	**0.000**	7.71	±0.96	3.92	±1.06	35	**0.026**
4-hydroxybenzoic acid	1635	8.09	±0.64	3.77	±0.28	14	**0.000**	3.92	±0.29	3.55	±0.55	63	0.531
3-HDAA	1658	1.07	±0.07	0.53	±0.05	18	**0.000**	0.59	±0.05	0.43	±0.08	42	0.071
4-hydroxydihydrocinnamyl alcohol	1687	0.07	±0.02	0.22	±0.04	89	**0.049**	0.22	±0.05	0.22	±0.09	61	0.461
9-ODA	1714	252.45	±20.15	125.16	±11.30	23	**0.000**	144.70	±13.18	95.84	±16.94	35	**0.026**
HVA	1716	0.51	±0.04	0.29	±0.02	24	**0.000**	0.32	±0.02	0.24	±0.03	46	0.115
9-oxodecanoic acid	1750	0.77	±0.09	0.44	±0.04	53	**0.001**	0.51	±0.05	0.34	±0.04	32	**0.016**
4-hydroxy-3methoxybenzoic acid	1785	2.66	±0.21	1.48	±0.13	33	**0.000**	1.73	±0.14	1.12	±0.19	33	**0.019**
9-HDA	1798	37.65	±3.87	19.56	±1.68	35	**0.000**	22.91	±2.04	14.54	±2.12	32	**0.016**
Unidentified 2	1817	0.91	±0.09	0.63	±0.05	66	**0.006**	0.66	±0.07	0.58	±0.09	63	0.531
10-HDAA	1820	2.84	±0.23	1.57	±0.13	28	**0.000**	1.75	±0.15	1.30	±0.21	47	0.129
10-HDA	1873	34.87	±3.95	23.90	±2.63	79	**0.021**	24.64	±3.57	22.81	±4.04	73	0.935
Unidentified 3	1885	0.49	±0.04	0.32	±0.03	45	**0.000**	0.33	±0.02	0.29	±0.07	56	0.311
C10:0 DA	1907	0.17	±0.01	0.16	±0.02	130	0.532	0.19	±0.02	0.13	±0.02	47	0.129
Dihydroferulic acid	1907	0.30	±0.02	0.16	±0.01	19	**0.000**	0.18	±0.01	0.13	±0.02	41	0.062
Alkane 1	1918	2.17	±0.15	1.22	±0.10	31	**0.000**	1.40	±0.11	0.95	±0.17	65	0.605
C10:1 DA	1957	3.29	±0.32	2.35	±0.20	71	**0.009**	2.48	±0.23	2.16	±0.36	40	0.055
Unidentified 4	1986	2.86	±0.31	1.36	±0.16	43	**0.000**	1.59	±0.21	1.03	±0.24	28	**0.008**
Unidentified 5	2023	6.88	±0.68	4.31	±0.45	62	**0.003**	5.23	±0.52	2.92	±0.61	64	0.567
Palmitic acid	2052	0.79	±0.04	0.42	±0.03	14	**0.000**	0.39	±0.03	0.45	±0.06	45	0.103
Unidentified 6	2068	4.74	±0.40	3.48	±0.33	74	**0.013**	3.91	±0.43	2.83	±0.45	59	0.397
Alkane 2	2119	0.36	±0.03	0.27	±0.02	60	**0.003**	0.25	±0.02	0.29	±0.05	66	0.643
Octadecenoic acid C18:1	2022	7.55	±0.34	2.60	±0.35	10	**0.000**	2.94	±0.52	2.08	±0.35	69	0.765

All significant differences with p<0.05 are marked in bold.

Finally, we compared the relative proportions (compared to the total gland quantity) of the 27 individual compounds between virgin and mated queens, and between SDI and MDI queens (Table 2). The relative proportion of 13 compounds was significantly different between virgins and mated queens, and 10 of these compounds were significantly higher in mated queen mandibular glands. There were fewer compounds with significantly different relative proportions between SDI and MDI queens. Two compounds (8-hydroxyoctanoic acid and unidentified 5) were present in significantly higher proportions in SDI queens than in MDI queens (Table 2), while six compounds (4-hydroxybenzoic acid, unidentified 2, (*E*)-dec-2-enedioic acid, palmitic acid, alkane 2 and stearic acid) were significantly higher in MDI than in SDI queens mandibular glands (Table 2). Of the QMP components, relative levels of 9-ODA were significantly lower in mated queens compared to virgins, while HOB levels were significantly higher. None of the QMP components differed between SDI and MDI queens.

**Table 2:** Chemical identity and relative proportion (mean±STD) of the queen mandibular gland of honey bees *Apis mellifera* belonging to different queen group: virgin, single (SDI) and multi (MDI) drone inseminated queens.

	Retention Index	Virgin (N = 12)		Inseminated (n = 25)		Mann Whitney (Virgin vs Inseminated)		SDI (n = 15)		MDI (n = 10)		Mann Whitney (SDI vs MDI)	
		mean	STD	mean	STD	*U*	p	mean	STD	mean	STD	*U*	p
Unidentified 1	1408	0.21	±0.05	0.42	±0.06	41	**0.000**	0.33	±0.03	0.57	±0.13	49	0.149
HOB	1499	0.06	±0.01	0.10	±0.01	50	**0.001**	0.10	±0.01	0.10	±0.02	74	0.956
4-hydroxy-3methoxybenzoic acid	1512	1.19	±0.07	1.15	±0.08	124	0.399	1.24	±0.12	1.05	±0.09	62	0.471
8-HOAA	1627	3.08	±0.12	2.79	±0.26	113	0.230	3.39	±0.29	2.01	±0.36	31	**0.015**
4-hydroxybenzoic acid	1635	2.11	±0.10	1.97	±0.12	110	0.194	1.78	±0.10	2.31	±0.21	35	**0.027**
3-HDAA	1658	0.28	±0.02	0.28	±0.02	113	0.230	0.26	±0.03	0.29	±0.04	72	0.868
4-hydroxydihydrocinnamyl alcohol	1687	0.02	±0.00	0.12	±0.03	37	**0.000**	0.10	±0.02	0.16	±0.07	70	0.782
9-ODA	1714	64.77	±0.63	60.38	±0.96	73	**0.012**	61.19	±1.27	58.65	±1.36	49	0.149
HVA	1716	0.13	±0.01	0.15	±0.01	106	0.153	0.14	±0.00	0.16	±0.01	50	0.166
9-oxodecanoic acid	1750	0.19	±0.01	0.23	±0.01	84	**0.032**	0.22	±0.01	0.24	±0.03	71	0.824
4-hydroxy-3methoxybenzoic acid	1785	0.68	±0.01	0.72	±0.02	104	0.136	0.76	±0.02	0.68	±0.03	47	0.120
9-HDA	1798	9.41	±0.40	9.70	±0.28	136	0.650	9.85	±0.34	9.43	±0.50	63	0.506
Unidentified 2	1817	0.23	±0.01	0.32	±0.02	52	**0.001**	0.29	±0.02	0.37	±0.03	31	**0.015**
10-HDAA	1820	0.72	±0.02	0.77	±0.03	128	0.475	0.77	±0.04	0.80	±0.06	63	0.506
10-HDA	1873	8.62	±0.56	12.04	±1.01	95	0.074	10.96	±1.28	14.11	±1.48	41	0.059
Unidentified 3	1885	0.13	±0.00	0.16	±0.02	67	**0.007**	0.15	±0.00	0.19	±0.04	60	0.405
C10:0 DA	1907	0.04	±0.00	0.08	±0.01	38	**0.000**	0.09	±0.01	0.08	±0.01	74	0.956
Dihydroferulic acid	1907	0.08	±0.00	0.08	±0.00	114	0.243	0.08	±0.00	0.08	±0.00	53	0.222
Alkane 1	1918	0.57	±0.02	0.60	±0.01	113	0.230	0.61	±0.02	0.57	±0.02	54	0.244
C10:1 DA	1957	0.84	±0.03	1.17	±0.06	32	**0.000**	1.09	±0.06	1.32	±0.10	37	**0.035**
Unidentified 4	1986	0.71	±0.04	0.68	±0.06	118	0.299	0.69	±0.09	0.63	±0.06	73	0.912
Unidentified 5	2023	1.75	±0.07	2.05	±0.11	99	0.098	2.31	±0.15	1.72	±0.11	30	**0.013**
Palmitic acid	2052	0.22	±0.02	0.26	±0.04	130	0.516	0.18	±0.02	0.37	±0.09	39	**0.046**
Unidentified 6	2068	1.21	±0.04	1.78	±0.11	59	**0.003**	1.76	±0.16	1.86	±0.16	62	0.471
Alkane 2	2119	0.10	±0.01	0.15	±0.03	72	**0.011**	0.12	±0.01	0.21	±0.06	35	**0.027**
Octadecenoic acid C18:1	2022	2.11	±0.23	1.42	±0.19	64	**0.005**	1.20	±0.26	1.50	±0.29	58	0.346
Stearic acid	2249	0.54	±0.05	0.45	±0.05	85	**0.035**	0.34	±0.06	0.56	±0.09	33	**0.020**

All significant differences with p<0.05 are marked in bold. (*E*)-4-hydroxycinnamic acid (1948), (*E*)-coniferyl alcohol (1957), traces

##### Brain gene expression levels of *Amfor*

**Figure 4:**
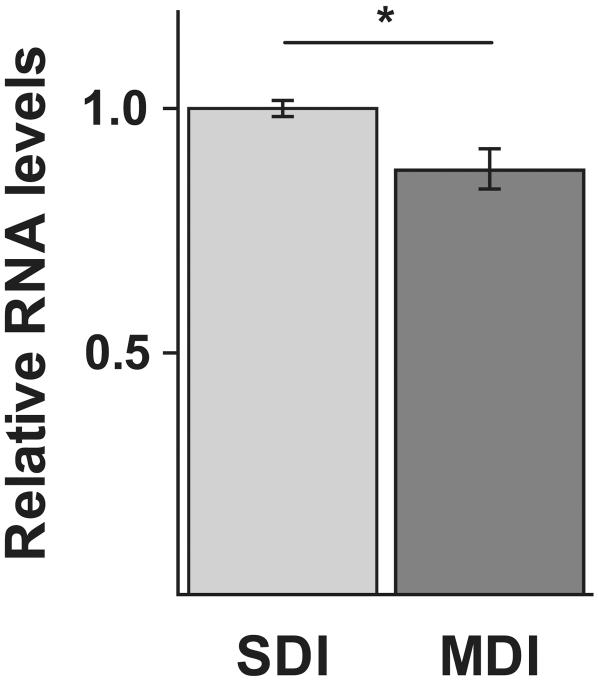
Brain gene expression levels are affected by insemination quantity. Expression levels of *Amfor* were monitored using quantitative real-time PCR using individual brains (SDI: N = 9; MDI: N = 9). SDI queens have significantly higher levels of *Amfor* than MDI queens (U = 10, p = 0.022). Data represent mean values, −/+ standard error (converted to the same arbitrary scale as the mean), relative to the SDI brains.

We used quantitative real-time PCR to measure *Amfor* expression levels in the queens' brains to determine if insemination treatment altered brain gene expression and therefore presumably altered neuronal properties in the brain. *Amfor* expression was significantly higher in SDI queens than in MDI queens (U = 10, p = 0.02; Figure 4).

## Discussion

In this study, we instrumentally inseminated queen honey bees to determine if insemination quantity alters their physiology and social interactions under controlled environmental conditions. Our results clearly demonstrate that insemination quantity alters queen physiology, queen pheromone profiles, and queen-worker interactions. MDI queens elicited a stronger retinue than SDI queens in natural colony conditions, and their mandibular gland extracts were more attractive in preference assays with caged worker bees. Moreover, GC-MS analysis of the mandibular gland chemical profiles revealed significant differences between SDI and MDI queens. Finally, brain-expression levels of a behaviorally relevant gene were significantly different between the SDI and MDI queens. All of these results suggest that insemination quantity can have profound effects on queen physiology and behaviour.

Comparisons of the mandibular gland profiles between MDI and SDI queens may lead to the identification of new components of queen pheromone. Previously, studies focusing on compounds that elicited a retinue response identified the five-component QMP blend [18]. Another study identified four additional compounds that did not elicit a retinue on their own, but synergized with QMP to produce a stronger retinue response [17]. However, the retinue response was still not as strong as that elicited by the live queens, suggesting that additional active compounds may still exist. In this study, we found that MDI queens elicited a larger retinue than SDI queens in observation hive colonies. Since the mandibular glands are the main source of QMP, we tested the mandibular gland extracts of virgin, SDI, and MDI queens on worker retinue responses in cages. When tested individually, the glands were equally attractive, though all glands are qualitatively comparable and contained the main QMP compounds [18]. However, when the glands were compared in a choice test, there were clear differences. Indeed, the glands of the inseminated queens were more attractive than gland of virgins, while the gland extracts of MDI queens were more attractive than gland of SDI queens. This suggests that MDI queen mandibular gland extracts have additional compounds that synergize with 9-ODA and 9-HDA to make these extracts more attractive in a preference assay and that the relative proportions of the entire gland chemical profile affect the retinue response. Indeed, when the extracts were analyzed with GC-MS, the chemical profiles of the mandibular glands of the inseminated queens were significantly different from those of the virgins, and those of the MDI queens were significantly different from those of the SDI queens.

Our results suggest that these changes in pheromone profiles occur within days after insemination, and that additional changes occur over a long period of time. Previous studies have compared the queen mandibular gland compounds between 6-day-old virgin and 1-year-old mated laying queens, and mated queens queens had higher levels of 9-ODA, 9-HDA, HOB, HVA, and 10-HDAA, and lower levels of 10-HDA [14]. However, when virgins and mated queens of more similar ages were compared, the differences were less robust [13]. In particular, 12-day-old virgins and mated queens that were laying for only 1 day did not differ significantly between levels of 9-ODA, 9-HDA, HOB, or HVA. In our study, virgins and inseminated queens were age-matched, and all queens were collected 5 days after insemination and were not laying eggs. However, we still observed significant differences in the overall chemical profiles and in several individual compounds, suggesting that insemination quantity causes changes in pheromone production within days. Thus, our results suggest that changes in mandibular gland profiles occur immediately or shortly after insemination, but additional modifications occur over time that may be associated with age, egg laying, or both. Furthermore, the observation hives assays with live queens and the cage studies with queen mandibular gland extract demonstrate that workers can detect these differences in pheromone profiles and respond differently to virgins versus newly inseminated queens, and singly versus multiply inseminated queens.

Expression levels of a gene associated with phototaxis are also significantly altered by insemination quantity, suggesting that insemination quantity exerts effects of neuronal properties in the brain and possibly behavior. We focused our analysis on brain expression levels of the *foraging* gene (*Amfor;* GenBank Accession AF469010). Previous studies found that expression of *Amfor* is significantly higher in forager bees than in-hive bees [35]. Moreover, *Amfor* encodes a cGMP-dependent protein kinase (*PKG*) and treatment with a cGMP analog stimulates foraging behavior [35] and increases phototaxis [36]. Our results demonstrate that there are significant differences in gene expression levels in brains of SDI and MDI queens, and these differences may be associated with changes in flight and phototaxis, though behavioural studies are clearly necessary to determine if this is the case. However, previous work demonstrates that queens inseminated with lower quantities of semen (<8 µL) have an increased likelihood to attempt mating flights [37].

Clearly, insemination quantity has profound effects on queens, but what are the mechanisms by which insemination quantity triggers these changes in queen honey bees? As noted previously, a number of factors might play a role. The number of stored sperm may modulate queen post-mating physiology and behavior [38]. It is highly unlikely that queens can directly assess the number of sperm in their spermathecae since there is no nerve enervation into the spermathecae and the membranes of their spermathecae are rigid [39], [40], so there is likely to be some other mechanism—one that is possibly correlated with stored sperm number—that serves as the basis for these physiological and behavioral changes. One possible explanation is that insemination volume may be the cue that triggers these changes. As queens are inseminated with numerous drones, the large quantity of semen is temporarily stored in their lateral and median oviducts for several hours before a proportion (5–10%) of each male's sperm migrates into the spermathecae and the excess semen is excreted. Indeed, preliminary studies (Richard, Tarpy, Grozinger, unpublished data) suggest insemination volume may be a key contributor to the observed post-insemination changes. One possible mechanism to induce these changes is that queens may have stretch receptors in their oviducts that provide a negative stimulus for additional mating behavior. Similar mechanisms exist in other insect systems [41]–[43], and abdomen distension has been tested in honey bees [44], but it remains unclear if abdominal enlargement is a physiological mechanism for reproductive senescence in queen bees. Alternatively, queens may use a substance in their mates' ejaculate, such as a hormonal precursor or seminal protein that causes physiological changes and decreases their subsequent reproductive behavior. Male seminal proteins have shown to have significant inhibitory effects on mating in female *Drosophila* [45]–[52] and possibly bumble bees [53], [54], and several substances in drone honey bee ejaculate have been identified, including sugars, metal ions, and proteins [55]–[57]. Further studies will be necessary to determine which factor(s) is involved in causing the differences in queen pheromone profile and brain gene expression that we have observed between SDI and MDI queens.

Previous studies have demonstrated that there may be colony-level adaptive benefits for genetically diverse workers, in terms of increased resistance to disease [5], [58], homozygosity at the sex determination locus [59], or better regulation of colony division of labor [60], [61]. Here, we demonstrate that insemination quantity, and thereby potentially mating number, could also affect different aspects of queen physiology or quality that could affect colony fitness. Additional research will be necessary to see if the physiological and pheromonal differences of differentially inseminated or mated queens are used by the workers as honest signals of their insemination quality or fertility [62]–[64].

## Materials and Methods

### Bee rearing

Colonies headed by single-drone inseminated queens (*Apis mellifera carnica,* Glenn Apiaries, CA) were maintained at the NCSU Lake Wheeler Honey Bee Research Facility (Raleigh, NC). These colonies were used as genetic sources for producing supersister queens for the experiments. Due to the haplodiploid genome of the hymenoptera, the progeny of SDI-queens have a genetic relatedness of *G* = 0.75. Additionally, colonies headed by naturally mated *Apis mellifera ligustica* or Buckfast-SMR queens (B. Weaver, TX) were used to provide workers for the observation-hive and cage experiments. Note that while worker bees were derived from different source colonies for convenience, each individual experiment used workers from a single colony when testing differential responses to SDI or MDI queens and/or pheromone extract. Thus, though there might be differences between colonies in worker responses to queen pheromones (i.e., reference 65), this should not be a factor in our results.

### Queen rearing

Supersister queens were produced by grafting young larvae (<24 h) from a single source colony and reared as queens in a queenless colony [32]. Once all queen cells were capped, they were transferred to a dark incubator at 33°C and ∼40% relative humidity. One to two days prior to emergence, each capped queen cell was placed into an individual Plexiglas cage (10×10×7 cm). Frames of brood were removed from a hive and incubated at 33°C. Day-old workers were collected from the brood frames and 100 adult worker bees were placed in the cages with the capped queen cell. Bees were provided with water, food (45% honey/45% pollen/10% water), and a solution of 50% sucrose, and all cages were maintained in an incubator (at 33°C and ∼40% RH). The food was changed every two days. Five days after emergence, queens destined for insemination were treated with CO_2_ for 4.0 minutes [66], while virgins were handled but left untreated, since we wanted a baseline nonreproductive control group and CO_2_ treatment accelerates the transition to egg-laying [66]. Seven days after emergence, queens were again treated with CO_2_ for 4.0 minutes, during which time they were inseminated with semen from either one drone (single-drone inseminated treatment group, or SDI) or 10 brother drones (multi-drone inseminated treatment group, or MDI) by following standard insemination protocols which readily produce laying queens [32], though in our experiments queens were reared in cages without honeycomb, so there was no opportunities for them to lay eggs. The average drone produces approximately 1 µl of semen, thus SDI queens were inseminated with approximately 1 µl and MDI queens were inseminated with approximately 10 µl. Queens were then returned to their respective cages and collected 5 days after insemination onto dry ice and stored at 80°C. A total of 12 virgin, 15 SDI and 11 MDI queens were collected. Queens were reared in three separate cohorts (Cohorts 1∶ 5 SDI and 4 MDI; Cohorts 2∶ 5 SDI and 2 MDI and Cohorts 3∶ 12 virgin, 5 SDI and 5 MDI), but queens from different cohorts were combined for the subsequent chemical analysis and behavioural assays.

Prior to dissection, queen heads were removed and partially lyophilized to facilitate dissection [67], after which their mandibular glands and brains were dissected out on dry ice and stored for future processing. In order to verify insemination success, we counted the number of sperm in each spermatheca (SDI, N = 15 and MDI, N = 8). As would be expected, spermathecae of MDI queens (65*10^4^±25*10^4^ sperm) contained significantly more sperm than those of SDI queens (15*10^4^±8*10^4^ sperm; U = 20.0, p = 0.0098). Note that these quantities of sperm are lower than typical for inseminated queens, but the lack of comb, the presence of a small number of workers, and the small size of the cages may have minimize sperm storage [38].

### Behavioral assays

#### Observation hives

Supersister queens were produced as described above. Capped queen cells were removed from the queen-rearing colony and individually placed into 1.5 frame observation hives (34″×5″×21.5″ LxWxH). The entrance to each hive was blocked with queen excluder material so the queens could not exit the hives to take mating flights. Observation hives were stocked with ∼1000 one-day-old bees and a half-frame of honey/pollen. The observation hives were stored in a 33°C incubator for 3 days (to ensure that workers were old enough to properly thermoregulate) prior to being placed in a room with access to the outside via a window. As the queens emerged, individual plastic numbered tags (Thorne Ltd, UK) were glued to their thoraxes. Approximately five days after emergence, queens were captured and treated with carbon dioxide for 4.0 minutes. Two days later, queens were captured again and treated with carbon dioxide for 4.0 minutes during their insemination procedure. As outlined above, queens were inseminated with semen from either a single drone (single-drone inseminated, SDI) or 10 brother drones (multi-drone inseminated, MDI). Queens were then released back into their respective hives. One hour later, the number of worker bees in the queen's retinue (surrounding the queen, licking and antennating her) was counted every minute over a period of 5.0 minutes [17], [18]. One hour later, the number of workers contacting the queen was counted again. The retinue size was calculated by summing across all of the observations, as in previous studies [17], [68]. The retinue was monitored every day, twice a day, for five days. In total, 2 SDI and 2 MDI queens were assayed in 2005 and 5 SDI and 3 MDI in 2006.

Differences in the retinue size for SDI and MDI queens in observation hives were analyzed using a mixed-model ANOVA (PROC mixed in SAS, Cary, NC), with treatment and day viewed as fixed factors, while trial, trial*treatment and replicate (trial*treatment) were random effects. Thus ‘day’ was treated as a split plot factor.

#### Cage assays

We next compared worker responses to mandibular gland extracts of virgin, SDI, and MDI queens. The glands were extracted from each supersister queen produced as outlined above.

Frames of brood were removed from a hive and incubated at 33°C. Day-old workers were collected from the brood frames and 35 bees were placed in Plexiglas cages (10×10×7 cm). Bees were provisioned as described above. The cages were kept in a 33°C incubator with ∼40% relative humidity, and manipulations and observations were performed under red light to negate any potential behavioural effects. Cages were maintained for five days.

##### Experiment 1: Retinue response

The retinue response was monitored as described previously [17], [21], [68], [69]. Extracts of mandibular glands of individual queens were produced as described below (see chemical analysis section). 0.05 queen equivalents (Qeq) of queen mandibular gland extract from individual queens (Virgin: n = 3; SDI: n = 8; MDI: n = 7) was placed onto a glass coverslip, the solvent was allowed to evaporate, and the coverslip was then placed inside the cage at the same time every day for five days. The number of bees antennating and licking the coverslip was counted in each cage (“retinue”). The retinue was recorded 5, 10, and 15 minutes after pheromone introduction each day of the 5-day time course. A single trial was performed.

##### Experiment 2: Preference assay

This experiment was repeated twice (Trial 1: Fall 2005 and Trial 2: Spring 2006). Bees were exposed to 0.1 Qeq of synthetic QMP (Pherotech, Canada). Every day, 10 µl of QMP was placed on a microscope slide and allowed to evaporate before being placed in the cage. This amount of QMP mimics a live queen in assays of worker behaviour and physiology [70]. Since worker maturation is altered in the absence of queen pheromone [67], [68], [71], we reared these workers with QMP to mimic natural colony conditions. On the fifth day of the experiment, workers were presented with two slides containing equal quantities of extract (0.05 Qeq) from either a virgin and SDI queen (Trial 1: n = 9 and Trial 2: n = 10), a virgin and MDI queen (Trial 1: n = 10 and Trial 2: n = 10), or a SDI and MDI queen (Trial 1: n = 11 and Trial 2: n = 10). The number of workers contacting each slide was counted every 5.0 minutes for 15 minutes after slide presentation. Note that a total of 6 virgin, 6 SDI, and 6 MDI queens were used in this analysis. Queens were derived from 2 cohorts. Trial 1 and trial 2 were not significantly different, so the data were pooled for the subsequent analysis.

Data are presented as mean±SEM. The effect of the mated number was evaluated with a non-parametric Kruskall-Wallis ANOVA on ranks for global comparison. The worker preferences between two slides were evaluated with a parametric *t*-test.

### Chemical analysis

The mandibular glands were dissected and immersed in 50 µl of diethyl ether containing 0.4 µg/µl of undec-10-enoic acid (as an internal standard) for minimum of 24 hours. A 5 µl portion (approx. 0.1 bee equivalents) of an extract was placed in a small glass insert and the solvent gently evaporated. The residue was sylylated overnight at room temperature in the insert with 10 µl of neat bistrimethylsilyltrifluoroacetamide (BSTFA) [17]. The derivatized sample was diluted with hexane (100 µl) and a 2 µl portion was analyzed using gas chromatography on a HP 5890 equipped with capillary column (30 m×0.25 mm ID. 0.5 µm film thickness) DB-5 (5% diphenyl-95% dimethylsiloxane) column (J&W scientific, Folsom, CA) in splitless mode. Helium was used as the carrier gas at a head pressure of 18 psi (flow rate = 1.3 ml/min). The GC temperature was held at 100°C for 1 min and then increased at 5°C/min to 200°C (5 min), followed by an increase of 10°C/min to 250°C (15 min). Injector and FID temperatures were both set at 250°C. We extracted the mandibular gland of 12 virgin queens, 15 SDI queens and 10 MDI queens. All queens used for chemical analysis were raised from the same grafting source to reduce any genetic variation in their pheromone profiles. However, the queens were reared in three different cohorts.

Compound identification was achieved by splitless capillary gas-chromatography-mass spectrometry using a Hewlett-Packard 6890 GC and a model 5973A msd with an electron impact ion source and a HP-5ms capillary column (30 m×0.25 mm ID×0.25 µm film thickness).

To examine differences in profiles related to number of inseminations based on the relative proportion of the chemical compounds, a stepwise discriminant analysis was employed, using all the chemical compounds (Statistica 6.0. StatSoft® Inc.). A MANOVA test was also used to compare treatment effects (queen insemination quantity). As the MANOVA test was significant, we compared each compound using a univariate analysis on the relative proportion for each compound. Both parametric and non-parametric tests were used for individual component comparisons, and both tests revealed comparable statistic p value. Here, we present the results from the Mann-Whitney test.

The following abbreviations are used for chemicals found in the analysis: methyl *p*-hydroxybenzoate (HOB), 8-hydroxyoctanoitic acid (8-HOAA), 4-hydroxy-3-methoxyphenylethanol (HVA), *(E)*-9-oxodec-2-enoic acid (ODA), *(E)*-9-hydroxydec-2-enoic acid (9-HDA), 10-hydroxydecanoic acid (10-HDAA), *(E)*-10-hydroxydec-2-enoic acid (10-HDA), decanedioic acid (C10∶0 DA) and *(E)*-dec-2-enedioic acid (C10∶1 DA)

### Brain gene expression levels of *Amfor*

Total RNA was isolated from dissected brains using a RNeasy RNA extraction kit (Qiagen, Valencia CA), yielding 0.6-1.5 µg/brain. cDNA was synthesized from 150 ng RNA using Arrayscript reverse transcriptase (Ambion, CA). Expression levels of *Amfor* were measured using quantitative real-time RT-PCR (qRT-PCR) with an ABI Prism 7900 sequence detector and the SYBR green detection method (Applied Biosystems, Foster City, CA). *eIF3-S8*, a housekeeping gene that did not vary in expression levels in previous bee brain microarray studies [67], [72], was used as a loading control. For each sample, triplicate qRT-PCR reactions were performed and averaged. A standard curve was generated for each primer using dilutions of genomic DNA to calculate the relative quantities of mRNA levels for each sample. A dissociation curve and negative control (cDNA reaction without RT-enzyme) were used to ensure primer specificity and lack of contamination.

The sequences for the primers (5′ to 3′) used are as follows:

*Amfor* -F: AATATAACTTCCGGTGCAACGTATT;

*Amfor* -R: CGTTTGGATCACGGAAGAAAG;

*eIFS8*-F: TGAGTGTCTGCTATGGATTGCAA;

*eIFS8*-R: TCGCGGCTCGTGGTAAA;

We evaluated the brain gene-expression levels of 9 SDI queens and 9 MDI queens (derived from two cohorts). For each individual brain sample, the ratio of the expression level of *Amfor* to that of the control gene (*eIF3-S8*) was calculated.
